# Acute radial head replacement with bipolar prostheses: midterm results

**DOI:** 10.1007/s00590-020-02774-4

**Published:** 2020-08-31

**Authors:** Alessandro Nosenzo, Cristina Galavotti, Margherita Menozzi, Alice Garzia, Francesco Pogliacomi, Filippo Calderazzi

**Affiliations:** Department of Surgery, Orthopaedic Clinic, Parma University Hospital, via Gramsci 14, 43100 Parma, Italy

**Keywords:** Radial head, Elbow, Arthroplasty, Bipolar prostheses

## Abstract

In irreparable radial head fractures, especially if primary stabilizers of the elbow are damaged, the prosthetic replacement prevents instability and stiffness. Concerns have arisen over the use of bipolar press-fit prostheses due to the frequent finding of osteolysis and the risk of instability if compared to monopolar implants. Our aim was to assess midterm clinical and radiological outcomes of bipolar implants and the influence of osteolysis on proximal pain. Seventeen patients with irreparable fractures of the radial head, treated in acute with the same prosthetic model (rHEAD recon SBI/Stryker) between January 2015 and December 2018, were enrolled. Clinical assessment was performed using MEPS and DASH scores; a radiographic study was done to identify heterotopic ossifications and periprosthetic osteolysis. Outcomes at the last follow-up, according to MEPS, were excellent in 10 cases, good in 5 and fair in 2; none of the patients had severe pain or instability. In 3 cases, it was necessary to remove the implant, mainly because of early loosening. Radiological findings of osteolysis were detected in 9 cases, but no statistical correlation was found with MEPS and proximal pain. The use of bipolar implants is reliable if an accurate repair of ligament tears is performed and provides a good stability. Nevertheless, the risk of early aseptic loosening in uncemented implants is not negligible, and the follow-up of the patient must be strict. Late osteolysis does not seem to have clinical relevance, but further prospective studies are necessary to clarify this topic.

## Introduction

Radial head influence on the elbow stability as secondary stabilizer by resisting to valgus stress and on the prevention of posterolateral rotatory instability is well understood [1, 2]. When primary stabilizers such as the coronoid, lateral (LCL) and medial collateral ligaments (MCL) are damaged, the integrity of the radial head takes on a predominant role and must be preserved [3]; consequently, in comminuted and displaced fracture (Mason type III and Mason type IV), if an anatomical reconstruction is not a viable solution, the prosthetic replacement should always be performed [4–6]. Radial head arthroplasty allows the injured soft tissues acting as major stabilizers to heal properly by the restoration of the native radial length, decreases the edge loading thus preventing cartilage wear and the onset of osteoarthritis and reproduces radiocapitellar kinematics [7]. Modern radial head prostheses (RHP) try to re-establish the native radio-humeral contact during the entire range of motion in order to avoid stiffness, pain and capitellar erosion. Three different strategies have been adopted to achieve this goal; loose-fit stems with a self-centering mechanism that decreases the incongruences between the implant and capitellum/lesser sigmoid notch, RHP with anatomic radial head replicating as much as possible the native anatomy and bipolar RHP that allows a rotation between the neck and the head of 10–15 degrees in all the planes, thus solving the problem of the great variability of head–neck angles with an adaptive positioning of the implant. When they were first introduced by Judet in 1996, bipolar RHP had a long and cemented stem; conversely, more recent implants have anatomical and press-fit short stems.

Concerns have arisen over both the effect of the bipolarity on elbow stability that seems to be reduced if compared to monopolar implants and the wear of the polyethylene used between the stem and the radial head [8, 9]. Moreover, osteolysis with loosening of metallic press-fit stems seems to be a common finding but its clinical relevance is still unclear and controversial [10]. Our primary aim was to investigate the midterm clinical and radiological outcomes of bipolar prostheses in irreparable radial head fractures and subsequently to examine the impact of osteolysis both on proximal forearm pain and on the survival of implants.

## Materials and methods

This is a retrospective single-center study performed at the department of Orthopedic Surgery. A research in the surgical database with the following inclusion criteria was performed in December 2019: patient with irreparable acute fracture of the radial head, implant of a bipolar radial head prosthesis with short press-fit stem (rHEAD recon SBI/Stryker, Morrisville, PA, USA) performed by our senior author (F.C) between January 2015 and December 2018. Exclusion criteria were: previous ipsilateral surgery, chronic injuries. Twenty-two patients met the inclusion criteria, and demographic data were collected. Surgical records were analyzed by a trained Orthopaedic surgeon (C.G), investigating concomitant bony lesions and ligamentous injuries, use of cementation, associated surgical procedures and intra-operative complications such as massive bleeding or residual instability of the elbow. The preoperative CT scans and plain radiographs were investigated separately by two residents in Orthopaedic Surgery (A.N/M.M) allowing to identify the pattern of radial head fracture according to modified Mason classification [11], presence of coronoid fracture or other patterns of ulnar fractures [12, 13]; any discordances were discussed and solved by the senior author (F.C). Patients were subsequently convened to the follow-up evaluation. A clinical assessment was performed using the Mayo Elbow Performance Score (MEPS) and The Disabilities of Arm, Shoulder and Hand score (DASH score); any further surgery following the primary implant was recorded. Afterward, a radiographic study with standardized anteroposterior, oblique and lateral views of the affected elbow was carried out and images were analyzed (C.G). Heterotopic ossifications were graded from I to IV using Ilahi and Gabel system [14], presence of osteolysis and radiographic loosening around the stem were assessed with Morrey grading scale (Table 1) [15]. Plain radiographs at the final follow-up were compared with radiographs performed at the last ordinary postsurgical follow-up in order to point out the evolution in time of osteolysis. Data were statistically analyzed with the commercial package IBM SPSS Statistics (IBM Corp., Armonk, NY, USA); statistical summaries were obtained for all the variables. Linear correlations using Pearson correlation coefficient were calculated to identify predictive factors of worse MEPS, range of motion and pain and predictive factors of instability (age, gender, side and type of fracture, associated lesions of the elbow, presence of heterotopic ossifications). A further Pearson correlation coefficient was calculated to point out a possible relationship between the grade of osteolysis and clinical outcomes (MEPS, DASH score, pain, instability, range of motion). The relationship was considered strong for *r* > 0.7 and *r* < − 0.7; the results were considered statistically significant for *p* value < 0.05. All procedures performed involving human participants were in accordance with the ethical standards of the Italian national bioethics committee, and the study was approved by the Regional Ethic Committee with number of protocol 11396.

**Table 1:** Morrey grading system of periprosthetic osteolysis and radiographic loosening [15]

Type	
0	Radiolucent line less than 1 mm thick and involving less than 50% of the interface
I	Radiolucent line at least 1 mm thick and involving less than 50% of the interface
II	Radiolucent line more than 1 mm thick and involving more than 50% of the interface
III	Radiolucent line more than 2 mm thick and around the entire interface
IV	Gross loosening

## Results

**Fig. 1:**
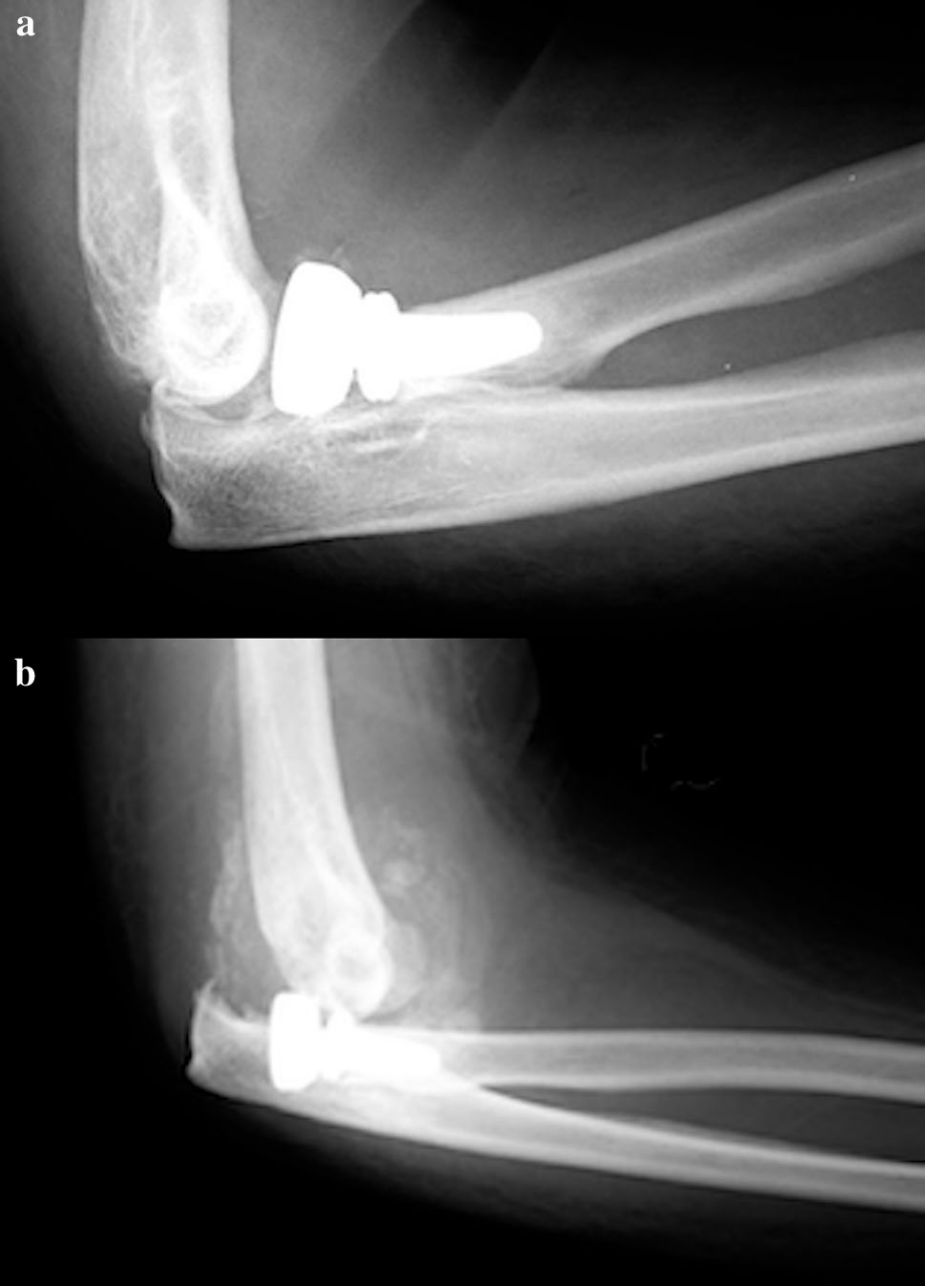
**a** A 71-year-old female patient treated with an uncemented prosthesis due to a terrible triad injury. **b** Dislocation of the prosthesis at 1 month with the need of dynamic hinged external fixation and removal of the implant. Presence of heterotopic ossification grade IV

Out of 22 patients that met the inclusion criteria, 5 were lost at the follow-up: Three were not traceable to the contact detail left and all other attempts were unsuccessful, whereas two did not give availability to further inspection. The final sample was made up of 17 patients, 10 males and 7 females with a mean age at the time of the surgery of 56 years (range 31–74 years). At preoperative plain radiographs, 9 radial head fractures were classified as Mason type IV and 8 as Mason type III with concomitant bony injuries in 11 cases (65%). According to surgical records, ligamentous injuries were detected in 10 cases and repaired in 8 cases (Table 2); the 17 rough-stem implants were press-fitted in 9 patients and fixed with cement in 8 cases when the fit was felt to be inadequate based on the assessment of the senior surgeon. The clinical results at the last follow-up (average 27.7 months, range 12–48) according to MEPS were excellent in 10 cases, good in 5 and fair in 2 with a mean MEPS of 91.2 points (range 70–100) and a mean DASH score of 6.6 points (range 0–34.1); average active flexion was 132° (range 105°–140°); average active extension deficit was 17° (range 0°–60°); average active pronation was 81° (range 10°–90°); average active supination was 74° (range 5°–90°). Twelve patients had no residual pain, 4 had mild, and only 1 had moderate proximal forearm pain. None had gross instability. Further surgery was necessary in 5 cases with an overall re-surgery rate of 29.4%. Due to severe proximal pain after surgery and radiological findings of early loosening of the stems, 2 press-fitted non-cemented bipolar RHPs were removed at 2 months, despite the surgeon had not reported any abnormalities during the primary operation. Because of elbow dislocation with gross instability, in 1 patient it was necessary the hardware removal and to place a dynamic hinged external fixator after considering advanced age and critical general conditions (Fig. 1). One patient, who sustained a terrible triad injury with concomitant fracture of humeral condyle, had the elbow dislocated at the ordinary follow-up at 1 month and was treated with a dynamic hinged external fixation. A case of terrible triad required a ligament revision with further suture anchors for both medial and lateral symptomatic instability. The overall implant removal rate was 17.6% (3/17) with no case of replacement of the implant or disassembling. At last follow-up, heterotopic ossifications were observed in 10 out of 17 patients (59%), classified as Ilahi–Gabel grade I in 2 cases, grade II in 4 and grade IV in 4. Radiological finding of osteolysis, visible as radiolucencies around the stem, was detected in 9 out of 14 cases (64%) excluding cases with the early removal of the prosthesis; according to Morrey, osteolysis was classified as type 0 in 5 patients, type I in 2, type II in 2, type III in 1 and type IV in 4. The temporal evolution of osteolysis between the postoperative follow-up, and the last follow-up is shown in Table 2; both patients with early aseptic loosening of the stem were classified as Morrey type IV at early postoperative follow-up. Statistical analysis with Pearson *R* test did not prove any strong positive or negative linear dependence between variables, finding no predictive factors of worse MEPS, pain or instability. Moreover, the amount of osteolysis showed just a weak negative linear correlation with MEPS (*R* = − 0.232) and a weak positive linear correlation with proximal forearm pain (*R* = 0.252) resulting in nonsignificant *p* value for confidence interval of 95%.

**Table 2:** Data of the patients

	Age	Sex	Side	Diagnosis	Ligament injury	Surgical procedure	Cementation	Second surgery	Postoperative follow-up (months)	Type of osteolysis at postoperative follow-up	Last follow-up (months)	Type of osteolysis at last follow-up	Heterotopic ossifications at last follow-up	Pain at last follow-up	MEPS at last follow-up	DASH at last follow-up
1	58	M	Right	Transolecranon fracture-dislocation	Yes	RHP + fixation of ulna + LCL repair	Yes	No	5	0	12	0	II	None	95	27.3
2	74	F	Left	Transolecranon fracture-dislocation	No	RHP + fixation of ulna	Yes	No	3	0	12	0	II	Mild	85	2.3
3	40	M	Left	Terrible triad + humeral condyle fracture	Yes	RHP + fixation of humeral condyle + fixation of coronoid + LCL repair + MCL repair	Yes	Positioning of dynamic hinged external fixator because of elbow dislocation	6	0	30	0	IV	None	95	2.3
4	58	M	Left	Transolecranon fracture-dislocation	No	RHP + fixation of ulna + fixation of coronoid	Yes	No	5	I	15	II	0	Mild	70	34.1
5	70	M	Left	Terrible triad	No	RHP	Yes	No	1	II	15	IV	0	None	100	6.8
6	67	F	Right	Terrible triad	Yes	RHP + LCL repair	Yes	No	4	I	36	I	0	None	100	0
7	42	F	Left	Isolated radial head fracture	No	RHP	No	Removal of the implant because of aseptic loosening	1	IV	24	–	0	Moderate	70	9
8	50	M	Left	Isolated radial head fracture	No	RHP	No	No	1	0	48	0	0	None	100	0
9	62	M	Right	Terrible triad	Yes	RHP + LCL repair	No	No	0	0	46	I	I	None	100	0
10	31	M	Left	Terrible triad	Yes	RHP + LCL repair + MCL repair	Yes	No	24	IV	45	IV	II	None	85	3.3
11	41	M	Left	Elbow dislocation with radial head fracture	Yes	RHP + LCL repair + MCL repair	No	No	2	III	21	IV	IV	None	95	4.5
12	68	F	Left	Monteggia-like lesion	No	RHP + fixation of ulna	No	No	4	0	20	III	I	Mild	85	9.1
13	48	F	Right	Terrible triad	Yes	RHP + LCL repair + MCL repair	No	Revision of LCL and MCL reconstruction because of symptomatic instability	3	0	19	0	IV	None	100	0
14	49	F	Left	Elbow dislocation with radial head fracture	Yes	RHP	No	No	1	0	48	IV	0	None	100	0
15	59	M	Right	Isolated radial head fracture	No	RHP	No	Removal of the implant because of aseptic loosening	1	IV	48	–	0	None	100	0
16	71	F	Right	Terrible triad	Yes	RHP	Yes	Positioning of dynamic hinged external fixator and removal of the implant because of elbow dislocation	3	0	12	–	IV	None	85	9.1
17	66	M	Right	Terrible triad	Yes	RHP + LCL repair + MCL repair	No	No	3	II	20	II	II	1	85	5

## Discussion

**Fig. 2:**
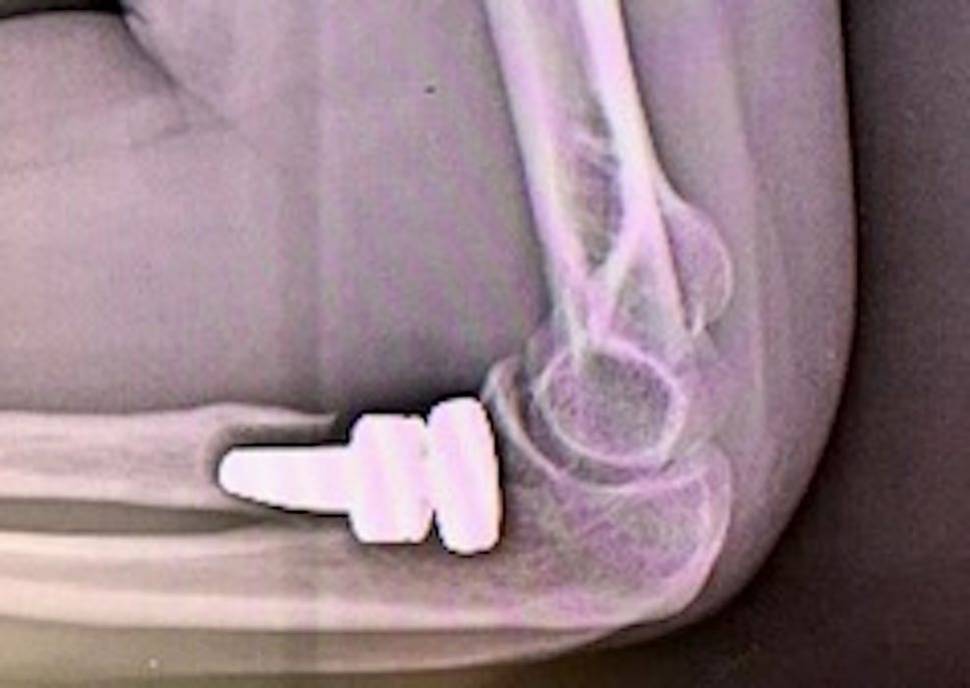
A case of aseptic loosening of the uncemented implant two months after surgery

**Fig. 3:**
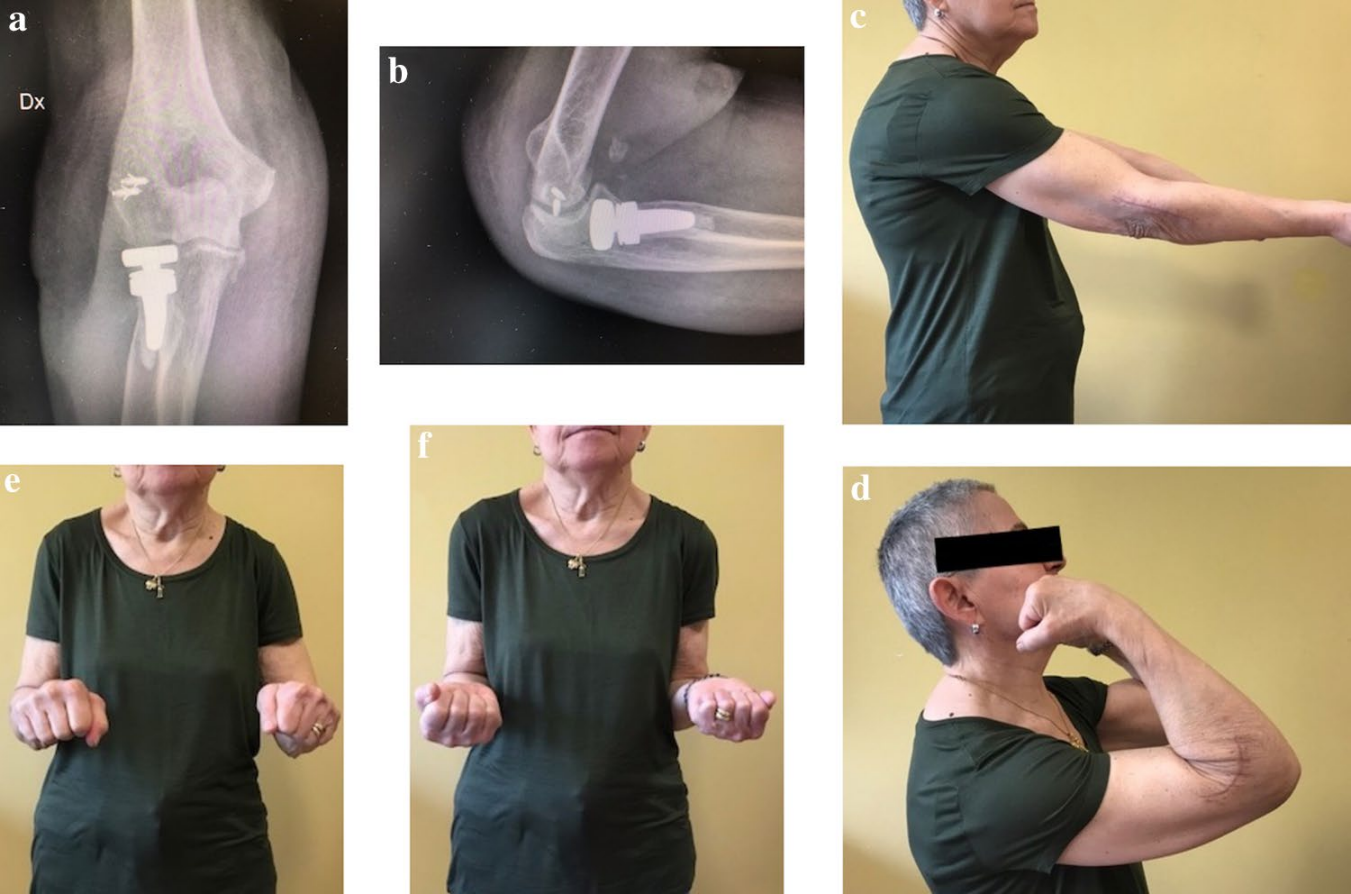
Four-month follow-up of a 67-year-old female patient (No. 6) treated with a cemented prosthesis and LCL repair with suture anchors due to a terrible triad injury. Radiological (**a**, **b**) and clinical assessment (**c**–**f**)

Biomechanical studies have shown the role of radial head in avoiding valgus and longitudinal instability, thus contributing to increase the rate of prosthetic replacement compared to simple radial head excision [2, 16, 17]. On the contrary, there is still little agreement on the reliability of several prosthetic devices that differ from each other for the features of the head (anatomic, non-anatomic, monopolar, bipolar), the design of the stem (loose-fit, press-fit, short or long), the type of fixation (cemented, uncemented) and the presence of modularity. Conflicting and controversial studies in the recent literature have consequences on daily surgical activity, making it difficult to choose a prosthetic model that ensures satisfactory clinical results, ease of implantation and low failures rate; this study originates from the need to evaluate outcomes, reliability and weaknesses of a single bipolar RHP (rHEAD recon SBI/Stryker, Morrisville, PA, USA), pointing out the rate of failure and the possible association between radiological signs and clinical findings. The theoretical advantage of bipolar prosthesis is a better radiocapitellar congruency due to the triaxial rotation of the head–neck system, thus decreasing the contact pressures on the capitulum during flexion–extension and prono-supination and the stress at the interface implant-bone during the forearm rotation; the prevention of the wear of the articular cartilage with exposure of subchondral capitellar bone could avoid persistent lateral forearm pain, a main cause of prosthetic revision [9]. Moreover, the association with a press-fit textured–surfaced stem is designed to facilitate the bone ingrowth with direct bone formation within the pores or apposition of bone from the adjacent bone tissue into the porous zone [18]. Our current study reports satisfactory midterm clinical results with good to excellent MEPS score in 15 out of 17 patients (88%) with just two patients reporting fair results and no one reporting poor results. Out of the two patients who showed a fair MEPS (70), one sustained a transolecranon fracture-dislocation treated with two plates on the ulna and a single-screw fixation for the coronoid process, and the other met an early aseptic loosening of an uncemented stem 2 months after the surgery; both reported a deficient prono-supination out of the functional range of motion. No instances of elbow gross instability were found at the follow-up; ligament concomitant injuries were detected in 10 patients and treated with suture anchors or direct suture in 8 cases. A single case of lateral collateral ligament (LCL) lesion in a terrible triad was not treated and the overstuffed implant dislocated at 1 month with the need of external fixation and removal of the prosthesis (Fig. 1). Biomechanical efficacy in restoration of elbow stability of monopolar and bipolar prostheses is still a topic of debate; for some authors, the variable coupling between bipolar prosthesis and capitellar surface opposes the translation forces in a worse way if compared to monopolar implants, thus leading to residual elbow instability [19]. Chanlalit et. al [8] reproduced in a cadaveric study on 8 fresh-frozen elbow specimens terrible triad injuries, demonstrating that monopolar RHP ensures greater radiocapitellar stability than bipolar RHP and that the anatomic shaped head is preferable over the non-anatomic; the subluxation force of the bipolar prosthesis was significantly less (1 +/− 1 N) than monopolar non-anatomic implant (12 +/− 1 N) and anatomic monopolar design (16 +/− 1 N), and the native radial head is statistically comparable to the latter (18 +/− 2 N). On the contrary, Hartzel el al. [20] assessed instability of monopolar and bipolar prostheses in a cadaveric study after terrible triad simulated injury followed by LCL reconstruction: No differences were found in improving valgus and external rotation laxity. In vivo studies seem to support this hypothesis; in the presence of an intact or appropriately repaired LCL the risk of instability is not increased with bipolar design and a residual laxity is not translated in clinically evident instability of the elbow [21–23]. Despite we reported satisfactory clinical outcomes, the need of second surgery in 5 out of 17 patients is not neglectable, with an overall rate of 29.4% and a removal rate of 17.6%, higher than that reported in the current literature. Indeed, in a systematic review and meta-analysis, Kachooei et al. [24] found a pooled rate of radial head prostheses removal or revision of 10%; more than half of the implants were revised for excision of heterotopic ossifications (47%) and for the treatment of elbow stiffness and limitation of the range of motion (42%); other relevant causes were pain (19%), aseptic loosening (16%), overstuffing (13%) and instability (12%). In our series, the primary indication for removal was an early aseptic symptomatic loosening of the prostheses; after two months both patients presented type IV radiolucencies surrounding uncemented stems (Fig. 2). Flinkillä et al. [10], in a paper on survival of press-fit stems on 37 cases, found a rate of symptomatic loosening of 26.5% with severe osteolysis in 5 patients and severe proximal forearm pain in 6 cases; the mean time elapsed between the primary surgery and loosening was 11 months. Compared to smooth-stemmed prostheses where stable mild radiolucencies around the stem seem to be a common finding [25], osteolysis in press-fit stems is more pronounced [26]. In a recent systematic review on failures of radial head arthroplasties, press-fit RHPs failed statistically more often because of symptomatic aseptic loosening compared to intentionally loose-fit RHPs, with radiolucencies around the stems occurring early after implantation [27]; furthermore, subcollar resorption due to stress shielding in press-fit stems is common but it is typically minor and non-progressive [28]. It has been proved that proper stem size in uncemented implants is critical in preventing periprosthetic osteolysis and aseptic loosening. A proper bony contact, through an appropriate intramedullary fit, decreases excessive micromotion that can reduce bone ingrowth leading to the formation of a layer of fibrous tissue [18, 29]. In our experience, we have noticed that it is quite difficult to obtain the optimal press-fit and to choose the proper stem size in uncemented implants. Indeed, although a feeling of significant stability with press-fit system is often reached in the operating room, we think that axial and shear stresses produced by bipolar RHP during common daily activities may lead to early stem mobilization and symptomatic osteolysis. For this reason, in the last implanted prostheses, we preferred to use cementation which provides immediate primary stability and avoids the risk of neck fractures due to the insertion of the maximum-sized rasp. Analyzing our failures, we believe it is appropriate to investigate patients showing early severe radiolucencies because it often correlates to symptomatic aseptic loosening. Despite we found no significant relationship between grade of osteolysis and poor outcomes at the final follow-up, it is advisable to submit recurring checks to the patient; indeed, the onset of proximal forearm pain in press-fit RHP is a strong indicator for loosening, even in the absence of radiographic signs [30]. Our study has several limitations. It is retrospective in nature; the number of cases is relatively small, and the heterogeneity of associated injuries and concomitant treatments could affect the statistical power of our investigation. We were not able to assess the exact progression of radiological parameters, because plain radiographs were not carried out routinely between the postsurgical follow-up and our final follow-up. In conclusion, we believe that the use of bipolar implants in complex elbow injuries can be considered reliable if an accurate treatment of injured soft tissues is performed and if an optimal primary stability of the stem is reached, even with the use of cementation if needed; indeed, the role of soft tissues repair is strongly underscored in the most recent literature as instability represents one of the main causes of implant revision [24]. Most of the time, instability is related to LCL complex failure and lateral ligaments suture in addition to the radial head prosthesis can translate into much better outcomes (Fig. 3). Besides, the patient surveillance must be strict, since most failures occur within three years. Whereas early severe radiolucency is often a sign of implant failure, clinical relevance of late and progressive osteolysis still remains uncertain, as our statistical analysis shows, providing weak and not significant correlations with outcomes; further prospective studies with more homogeneous cohorts of patients are necessary to clarify this topic.

## Data Availability

The authors confirm that the data supporting the findings of this study are available within the article. Further data that support the findings of this study are available from the corresponding author upon reasonable request.
